# Supplementary material for: Sex-Differences in the Pattern of Comorbidities, Functional Independence, and Mortality in Elderly Inpatients: Evidence from the RePoSI Register
Source: J Clin Med. 2019 Jan 12;8(1):81. doi: 10.3390/jcm8010081 (PMC6352226; doi:10.3390/jcm8010081)
Supplement: Supplementary File 1 [file jcm-08-00081-s001.pdf]

**APPENDIX: Investigators and co-authors of the REPOSI (REgistro POliterapie SIMI, Società Italiana di Medicina Interna) Study Group are as follows:**

***Steering Committee:*** Pier Mannuccio Mannucci *(Chair, Fondazione IRCCS Cà Granda Ospedale Maggiore Policlinico, Milano)*, Alessandro Nobili *(co-chair, Istituto di Ricerche Farmacologiche Mario Negri IRCCS, Milano),* Mauro Tettamanti, Luca Pasina, Carlotta Franchi *(Istituto di Ricerche Farmacologiche Mario Negri IRCCS, Milano),* Salvatore Corrao *(ARNAS Civico, Di Cristina, Benfratelli, DiBiMIS, Università di Palermo, Palermo),* Alessandra Marengoni *(Spedali Civili di Brescia, Brescia),* Francesco Salerno *(IRCCS Policlinico San Donato Milanese, Milano),* Matteo Cesari *(UO Geriatria, Università degli Studi di Milano),* Francesco Perticone *(Presidente SIMI),* Giuseppe Licata *(Azienda Ospedaliera Universitaria Policlinico P. Giaccone di Palermo, Palermo, Medicina Interna e Cardioangiologia),* Francesco Violi *(Policlinico Umberto I, Roma, Prima Clinica Medica),* Gino Roberto Corazza, *(Reparto 11, IRCCS Policlinico San Matteo di Pavia, Pavia, Clinica Medica I).*

***Clincal data monitoring and revision:*** Carlotta Franchi, Laura Cortesi *(Istituto di Ricerche Farmacologiche Mario Negri IRCCS, Milano).*

***Database Management and Statistics:*** Mauro Tettamanti, Laura Cortesi, Ilaria Ardoino *(Istituto di Ricerche Farmacologiche Mario Negri IRCCS, Milano).*

***Investigators:***
***Italian Hospitals***
Domenico Prisco, Elena Silvestri, Caterina Cenci, Giacomo Emmi *(Azienda Ospedaliero Universitaria Careggi Firenze, Medicina Interna Interdisciplinare);*
Gianni Biolo, Michela Zanetti, Martina Guadagni, Michele Zaccari (*Azienda Sanitaria Universitaria Integrata di Trieste, Clinica Medica Generale e Terapia Medica*);
Massimo Vanoli, Giulia Grignani, Edoardo Alessandro Pulixi *(Azienda Ospedaliera della Provincia di Lecco, Ospedale di Merate, Lecco, Medicina Interna);*
Mauro Bernardi, Silvia Li Bassi, Luca Santi, Giacomo Zaccherini *(Azienda Ospedaliera Policlinico Sant'Orsola-Malpighi, Bologna, Semeiotica Medica Bernardi);*
Elmo Mannarino, Graziana Lupattelli, Vanessa Bianconi, Francesco Paciullo *(Azienda Ospedaliera Santa Maria della Misericordia, Perugia, Medicina Interna);*
Ranuccio Nuti, Roberto Valenti, Martina Ruvio, Silvia Cappelli, Alberto Palazzuoli *(Azienda Ospedaliera Università Senese, Siena, Medicina Interna I);*
Oliviero Olivieri, Domenico Girelli, Thomas Matteazzi *(Azienda Ospedaliera Universitaria Integrata di Verona, Verona, Medicina Generale a indirizzo Immuno-Ematologico e Emocoagulativo);*
Mario Barbagallo, Ligia Dominguez, Floriana Cocita, Vincenza Beneduce, Lidia Plances *(Azienda Ospedaliera Universitaria Policlinico Giaccone Policlinico di Palermo, Palermo, Unità Operativa di Geriatria e Lungodegenza);*
Marco Zoli, Ilaria Lazzari, Mattia Brunori *(Azienda Ospedaliera Universitaria Policlinico S. Orsola-Malpighi, Bologna, Unità Operativa di Medicina Interna);*
Franco Laghi Pasini, Pier Leopoldo Capecchi, *(Azienda Ospedaliera Universitaria Senese, Siena, Unità Operativa Complessa Medicina 2);*
Giuseppe Palasciano, Maria Ester Modeo, Carla Di Gennaro *(Azienda Ospedaliero-Universitaria Consorziale Policlinico di Bari, Bari, Medicina Interna Ospedaliera "L. D'Agostino", Medicina Interna Universitaria "A. Murri");*
Maria Domenica Cappellini, Diletta Maira, Valeria Di Stefano, Giovanna Fabio, Sonia Seghezzi, Marta Mancarella *(Fondazione IRCCS Cà Granda Ospedale Maggiore Policlinico, Milano, Unità Operativa Medicina Interna IA);*
Matteo Cesari, Paolo Dionigi Rossi, Sarah Damanti, Marta Clerici, Federica Conti *(Fondazione IRCCS Cà Granda Ospedale Maggiore Policlinico, Milano, Geriatria);*
Gino Roberto Corazza, Emanuela Miceli, Marco Vincenzo Lenti, Martina Pisati, Costanza Caccia Dominioni *(Reparto 11, IRCCS Policlinico San Matteo di Pavia, Pavia, Clinica Medica I);*

Giovanni Murialdo, Alessio Marra, Federico Cattaneo, Roberto Pontremoli *(IRCCS Azienda Ospedaliera Universitaria San Martino-IST di Genova, Genova, Clinica di Medicina Interna 2);*
Maria Beatrice Secchi, Davide Ghelfi *(Ospedale Bassini di Cinisello Balsamo, Milano, Divisione Medicina);*
Luigi Anastasio, Lucia Sofia, Maria Carbone *(Ospedale Civile Jazzolino di Vibo Valentia, Vibo Valentia, Medicina interna);*
Francesco Cipollone, Maria Teresa Guagnano, Ermanno Angelucci, Emanuele Valeriani *(Ospedale Clinicizzato SS. Annunziata, Chieti, Clinica Medica);*
Gerardo Mancuso, Daniela Calipari, Mosè Bartone *(Ospedale Giovanni Paolo II Lamezia Terme, Catanzaro, Unità Operativa Complessa Medicina Interna);*
Giuseppe Delitala, Maria Berria *(Azienda ospedaliera-universitaria di Sassari, Clinica Medica);*
Maurizio Muscaritoli, Alessio Molfino, Enrico Petrillo *(Policlinico Umberto I, Sapienza Università di Roma, Medicina Interna e Nutrizione Clinica Policlinico Umberto I);*
Giuseppe Zuccalà, Gabriella D'Aurizio *(Policlinico Universitario A. Gemelli, Roma, Roma, Unità Operativa Complessa Medicina d'Urgenza e Pronto Soccorso)*
Giuseppe Romanelli, Alessandra Marengoni, Alberto Zucchelli *(Spedali Civili di Brescia, Brescia, Geriatria);*
Antonio Picardi, Umberto Vespasiani Gentilucci, Paolo Gallo, Chiara Dell'Unto *(Università Campus Bio-Medico, Roma, Medicina Clinica-Epatologia);*
Giorgio Annoni, Maurizio Corsi, Giuseppe Bellelli, Sara Zazzetta, Paolo Mazzola, Hajnalka Szabo, Alessandra Bonfanti *(Università degli studi di Milano-Bicocca Ospedale S. Gerardo, Monza, Unità Operativa di Geriatria);*
Franco Arturi, Elena Succurro, Mariangela Rubino *(Università degli Studi Magna Grecia, Policlinico Mater Domini, Catanzaro, Unità Operativa Complessa di Medicina Interna);*
Maria Grazia Serra, Maria Antonietta Bleve *(Azienda Ospedaliera "Cardinale Panico" Tricase, Lecce, Unità Operativa Complessa Medicina);*
Laura Gasbarrone, Maria Rosaria Sajeva *(Azienda Ospedaliera Ospedale San Camillo Forlanini, Roma, Medicina Interna 1);*
Antonio Brucato, Silvia Ghidoni *(Azienda Ospedaliera Papa Giovanni XXIII, Bergamo, Medicina 1);*
Fabrizio Fabris, Irene Bertozzi, Giulia Bogoni, Maria Victoria Rabuini, Elisabetta Cosi *(Azienda Ospedaliera Università di Padova, Padova, Clinica Medica I);*
Roberto Manfredini, Fabio Fabbian, Benedetta Boari, Alfredo De Giorgi, Ruana Tiseo *(Azienda Ospedaliera - Universitaria Sant'Anna, Ferrara, Unità Operativa Clinica Medica);*
Giuseppe Paolisso, Maria Rosaria Rizzo, *(Azienda Ospedaliera Universitaria della Seconda Università degli Studi di Napoli, Napoli, VI Divisione di Medicina Interna e Malattie Nutrizionali dell'Invecchiamento);*
Claudio Borghi, Enrico Strocchi, Valeria De Sando, Ilenia Pareo *(Azienda Ospedaliera Universitaria Policlinico S. Orsola-Malpighi, Bologna, Unità Operativa di Medicina Interna Borghi);*
Carlo Sabbà, Francesco Saverio Vella, Patrizia Suppressa, Pasquale Agosti, Andrea Schilardi, Francesca Loparco *(Azienda Ospedaliero-Universitaria Consorziale Policlinico di Bari, Bari, Medicina Interna Universitaria C. Frugoni);*
Luigi Fenoglio, Christian Bracco, Alessia Valentina Giraudo *(Azienda Sanitaria Ospedaliera Santa Croce e Carle di Cuneo, Cuneo, S. C. Medicina Interna);*
Silvia Fargion, Giulia Periti, Marianna Porzio, Slivia Tiraboschi *(Fondazione IRCCS Cà Granda Ospedale Maggiore Policlinico, Milano, Medicina Interna 1B);*
Flora Peyvandi, Raffaella Rossio, Barbara Ferrari, Giulia Colombo *(Fondazione IRCCS Cà Granda Ospedale Maggiore Policlinico, Milano, Medicina Interna 2);*
Valter Monzani, Valeria Savojardo, Christian Folli, Giuliana Ceriani *(Fondazione IRCCS Cà Granda Ospedale Maggiore Policlinico, Milano, Medicina Interna Alta Intensità);*
Francesco Salerno, Giada Pallini *(IRCCS Policlinico San Donato e Università di Milano, San Donato Milanese, Medicina Interna);*
Franco Dallegri, Luciano Ottonello, Luca Liberale, Lara Caserza, Kassem Salam *(Università di Genova, Genova, Medicina Interna 1);*
Nicola Lucio Liberato, Tiziana Tognin (*ASST di Pavia, UOSD Medicina Interna, Ospedale di Casorate Primo, Pavia);*

Giovanni Battista Bianchi, Sabrina Giaquinto (*Ospedale "SS Gerosa e Capitanio" di Lovere, Bergamo, Unità Operativa Complessa di Medicina Generale, Azienda Ospedaliera "Bolognini" di Seriate, Bergamo);*

Francesco Purrello, Antonino Di Pino, Salvatore Piro *(Ospedale Garibaldi Nesima, Catania, Unità Operativa Complessa di Medicina Interna);*

Renzo Rozzini, Lina Falanga, Elena Spazzini, Camillo Ferrandina *(Ospedale Poliambulanza, Brescia, Medicina Interna e Geriatria);*

Giuseppe Montrucchio, Paolo Petitti *(Dipartimento di Scienze Mediche, Università di Torino, Città della Scienza e della Salute, Torino, Medicina Interna 2 U. Indirizzo d'Urgenza);*

Raffaella Salmi, Piergiorgio Gaudenzi *(Azienda Ospedaliera-Universitaria S. Anna, Ferrara, Unità Operativa di Medicina Ospedaliera II);*

Francesco Violi, Ludovica Perri *(Policlinico Umberto I, Roma, Prima Clinica Medica);*

Raffaele Landolfi, Massimo Montalto, Antonio Mirijello *(Policlinico Universitario A. Gemelli, Roma, Clinica Medica);*

Luigina Guasti, Luana Castiglioni, Andrea Maresca, Alessandro Squizzato, Marta Molaro, Alessandra Grossi *(Università degli Studi dell'Insubria, Ospedale di Circolo e Fondazione Macchi, Varese, Medicina Interna I);*

Marco Bertolotti, Chiara Mussi, Maria Vittoria Libbra, Giulia Dondi, Elisa Pellegrini, Lucia Carulli *(Università di Modena e Reggio Emilia, AUSL di Modena, Modena, Nuovo Ospedale Civile, Unità Operativa di Geriatria e U.O. di Medicina a indirizzo Metabolico Nutrizionistico);*

Francesco Perticone, Lidia Colangelo, Tania Falbo *(Università Magna Grecia Policlinico Mater Domini, Catanzaro, Unità Operativa Malattie Cardiovascolari Geriatriche);*

Vincenzo Stanghellini, Roberto De Giorgio, Eugenio Ruggeri, Sara del Vecchio *(Dipartimento di Scienze Mediche e Chirurgiche, Unità Operativa di Medicina Interna, Università degli Studi di Bologna/Azienda Ospedaliero-Universitaria S.Orsola-Malpighi, Bologna);*

Andrea Salvi, Roberto Leonardi, Giampaolo Damiani *(Spedali Civili di Brescia, U.O. 3a Medicina Generale);*

Armando Gabrielli, William Capeci, Massimo Mattioli, Giuseppe Pio Martino, Lorenzo Biondi, Pietro Pettinari *(Clinica Medica, Azienda Ospedaliera Universitaria - Ospedali Riuniti di Ancona);*

Riccardo Ghio, Anna Dal Col *(Azienda Ospedaliera Università San Martino, Genova, Medicina III);*

Salvatore Minisola, Luciano Colangelo *(Policlinico Umberto I, Roma, Medicina Interna F e Malattie Metaboliche dell'osso);*

Antonella Afeltra, Benedetta Marigliano, Maria Elena Pipita *(Policlinico Campus Biomedico Roma, Roma, Medicina Clinica);*

Pietro Castellino, Julien Blanco, Luca Zanoli, Samuele Pignataro (*Azienda Ospedaliera Universitaria Policlinico – V. Emanuele, Catania, Dipartimento di Medicina);*

Valter Saracco, Marisa Fogliati, Carlo Bussolino *(Ospedale Cardinal Massaia Asti, Medicina A);*

Francesca Mete, Miriam Gino *(Ospedale degli Infermi di Rivoli, Torino, Medicina Interna)*

Antonio Cittadini, Carlo Vigorito, Michele Arcopinto, Andrea Salzano, Emanuele Bobbio, Alberto Maria Marra, Domenico Sirico *(Azienda Policlinico Universitario Federico II di Napoli, Napoli, Medicina Interna e Riabilitazione Cardiologica);*

Guido Moreo, Francesca Gasparini, Silvia Prolo, Gloria Pina *(Clinica San Carlo Casa di Cura Polispecialistica, Paderno Dugnano, Milano, Unità Operativa di Medicina Interna);*

Alberto Ballestrero, Fabio Ferrando *(Clinica Di Medicina Interna ad Indirizzo Oncologico, Azienda Ospedaliera Università San Martino di Genova);*

Sergio Berra, Simonetta Dassi, Maria Cristina Nava *(Medicina Interna, Azienda Ospedaliera Guido Salvini, Garnagnate, Milano);*

Bruno Graziella, Stefano Baldassarre, Salvatore Fragapani, Gabriella Gruden *(Medicina Interna III, Ospedale S. Giovanni Battista Molinette, Torino);*

Giorgio Galanti, Gabriele Mascherini, Cristian Petri, Laura Stefani *(Agenzia di Medicina dello Sport, AOUC Careggi, Firenze);*

Margherita Girino, Valeria Piccinelli (Medicina Interna, Ospedale S. Spirito Casale Monferrato, Alessandria);

Francesco Nasso, Vincenza Gioffrè, Maria Pasquale *(Struttura Operativa Complessa di Medicina Interna, Ospedale Santa Maria degli Ungheresi, Reggio Calabria);*

Giuseppe Scattolin, Sergio Martinelli, Mauro Turrin *(Medicina Interna, Ospedale di Monselice, Padova);*

Leonardo Sechi, Cristina Catena, Gianluca Colussi *(Clinica Medica, Azienda Ospedaliera Universitaria, Udine).*
Nicola Passariello, Luca Rinaldi *(Presidio Medico di Marcianise, Napoli, Medicina Interna);*
Franco Berti, Giuseppe Famularo, Tarsitani Patrizia *(Azienda Ospedaliera San Camillo Forlanini, Roma, Medicina Interna II);*
Roberto Castello, Michela Pasino *(Ospedale Civile Maggiore Borgo Trento, Verona, Medicina Generale e Sezione di Decisione Clinica);*
Gian Paolo Ceda, Marcello Giuseppe Maggio, Simonetta Morganti, Andrea Artoni*(Azienda Ospedaliero Universitaria di Parma, U.O.C Clinica Geriatrica);*
Stefano Del Giacco, Davide Firinu, Francesca Losa, Giovanni Paoletti *(Policlinico Universitario Dulio Casula, Azienda Ospedaliero-Universitaria di Cagliari, Cagliari, Medicina Interna, Allergologia ed Immunologia Clinica);*
Giuseppe Montalto, Anna Licata, Valentina Malerba *(Azienda Ospedaliera Universitaria Policlinico Paolo Giaccone, Palermo, U.O.S Prevenzione Malattie Epatobiliari);*
Lasco Antonino, Giorgio Basile, Catalano Antonino *(Azienda Ospedaliera Universitaria Policlinico G. Martino, Messina, Unità Operativa di Geriatria);*
Lorenzo Malatino, Benedetta Stancanelli, Valentina Terranova, Salvatore Di Marca *(Azienda Ospedaliera per l'Emergenza Cannizzaro, Catania, (Clinica Medica Università di Catania);*
Patrizia Mecocci, Carmelinda Ruggiero, Virginia Boccardi *(Università degli Studi di Perugia-Azienda Ospedaliera S.M. della Misericordia, Perugia, Struttura Complessa di Geriatria);*
Tiziana Meschi, Fulvio Lauretani, Andrea Ticinesi *(Azienda Ospedaliera Universitaria di Parma, U.O Medicina Interna e Lungodegenza Critica);*
Pietro Minuz, Luigi Fondrieschi *(Azienda Ospedaliera Universitaria Verona, Policlinico GB Rossi, Verona, Medicina Generale per lo Studio ed il Trattamento dell'Ipertensione Arteriosa);*
Mario Pirisi, Gian Paolo Fra, Daniele Sola *(Azienda Ospedaliera Universitaria Maggiore della Carità, , Medicina Interna 1);*
Massimo Porta, Piero Riva *(Azienda Ospedaliera Universitaria Città della Salute e della Scienza di Torino, Medicina Interna 1U);*
Roberto Quadri *(Ospedale di Ciriè, ASL TO4, Torino, S.C. Medicina Interna);*
Giorgio Scanzi, Caterina Mengoli, Stella Provini, Laura Ricevuti *(ASST Lodi, Presidio di Codogno, Milano, Medicina);*
Emilio Simeone, Rosa Scurti, Fabio Tolloso *(Ospedale Spirito Santo di Pescara, Geriatria);*
Roberto Tarquini, Alice Valoriani, Silvia Dolenti, Giulia Vannini*(Ospedale San Giuseppe, Empoli, USL Toscana Centro, Firenze, Medicina Interna I);*
Alberto Tedeschi, Lucia Trotta *(ASST Fatebenefratelli - Sacco, Milano, Medicina Interna a indirizzo Pneumologico);*
Riccardo Volpi, Pietro Bocchi, Alessandro Vignali *(Azienda Ospedaliera Universitaria di Parma, Clinica e Terapia Medica).*
Sergio Harari, Chiara Lonati, Mara Cattaneo (*Ospedale San Giuseppe Multimedica Spa, U.O. Medicina Generale*)
